# Circulating exosomal lncRNAs as predictors of risk and unfavorable prognosis for large artery atherosclerotic stroke

**DOI:** 10.1002/ctm2.555

**Published:** 2021-12-19

**Authors:** Shuai Zhang, Xia Wang, Ruihua Yin, Qi Xiao, Yuanyuan Ding, Xiaoyan Zhu, Xudong Pan

**Affiliations:** ^1^ Department of Neurology The Affiliated Hospital of Qingdao University Qingdao China; ^2^ Department of Critical Care Medicine The Affiliated Hospital of Qingdao University Qingdao China

**Abbreviations**
AIS: acute ischemic stroke
AUC: Area Under Curve
GO: Gene Ontology
KEGG: Kyoto Encyclopedia of Genes and Genomes
LAA: large artery atherosclerotic
LDL: low‐density lipoprotein
lncRNA: long noncoding RNA
mRS: modified Rankin Scale
NIHSS: National Institutes of Health Stroke Scale
NRI: Net Reclassification Index
OR: odds ratio
ROC: receiver operating characteristic curve
SAO: small artery occlusion
TC: total cholesterol
TG: triglycerides
TOAST: Trial of Org 10172 in Acute Stroke Treatment

Dear Editor,

Large artery atherosclerotic (LAA) stroke has the worst prognosis and the heaviest burden among all stroke subtypes.^1^ A rapid and reliable diagnostic and prognostic biomarker is the key reference factor for early treatment strategies in LAA stroke management.^2^ Neuroimaging and clinical risk scores could evaluate the diagnosis and prognosis for LAA stroke. However, they still have some limitations.^3^ Exosomal lncRNAs, stable in peripheral blood, show promising diagnostic and prognostic value for cancer and other diseases.^4^, ^5^ Here, we revealed the diagnostic value and prognostic performance of exosomal lncRNAs in LAA stroke through compared the expression among different substyle stroke. More importantly, the differences between plasmatic lncRNAs and exosomal lncRNAs in expression and diagnostic performance were also compared.

To obtain differentially expressed exosomal lncRNAs in LAA stroke, 602 participants recruited from 2019 to 2021 at the Affiliated Hospital of Qingdao University in China. All patients were randomly assigned into discovery set (n = 12), validation set (n = 80) and replication set (n = 510) (Figure S1A, Table S1 in Supporting Information). Of 201 LAA stroke patients underwent NIHSS (defined as mild stroke: score ranged 0–6; moderate:7‐15; and severe: > 15) and mRS (defined as favorable outcome: score ranged 0 to 2; poor outcome: > 2), Chi‐square test showed NIHSS scores were different between favorable and unfavorable outcomes in LAA patients (*P *< 0.001, Table S2).^6^, ^7^ Plasma exosomes were extracted and identified by TEM, WB and NTA (Figures 1A‐C).^8^ The shape (elliptical concave shaped vesicles), positive markers (CD 9, CD 63 and TSG 101) and size (30 to 150 nm) of exosomes were consistent with the standard of MISEV issued by ISEV.^9^ RNA sequencing screened a total of 319 differentially expressed exosomal lncRNAs (222 downregulated and 97 upregulated) in discovery set (Figure S1B, Figure 1D). GO and KEGG pathway analysis of target genes of exosomal lncRNAs were mainly enriched in the pathological process of atherosclerosis (Figures S1C‐E).

**FIGURE 1:**
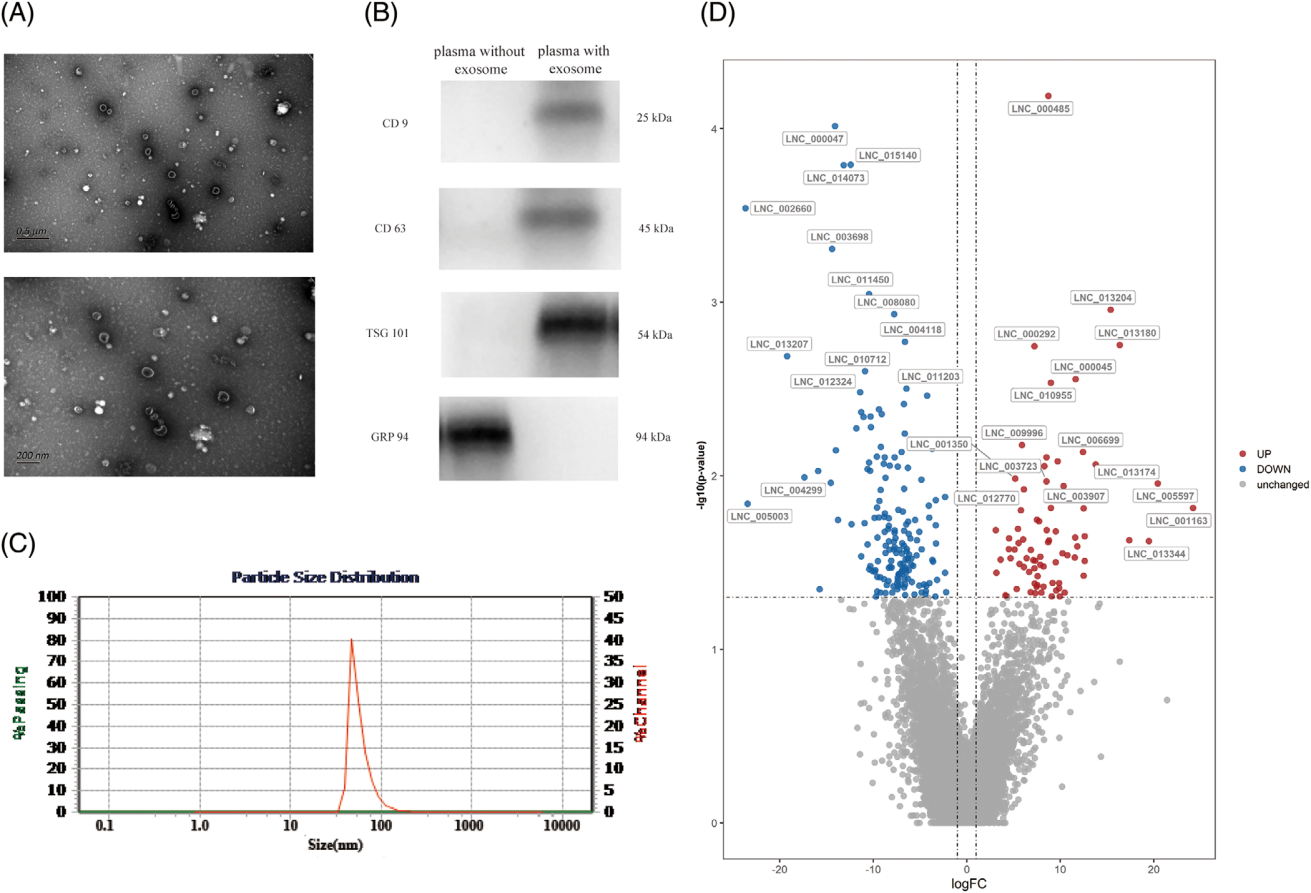
Identification of exosome and visualization of differentially expressed exosomal lncRNAs. (A) Exosomes were observed as elliptical concave shaped vesicles by TEM (up: Figure scale bar = 0.5μm; down: Figure scale bar = 200 nm). (B) CD9 (25 kDa) and TSG101 (54 kDa), CD63 (45 kDa) and GRP 94 (94 kDa) were analyzed by Western blotting. Lane left: plasma without exosome; lane right: plasma with exosome. (C) NTA revealed the sizes of exosomes were ranged from 30–150 nm in diameter (accounted for 95.42% of all particles). (D) Volcano plots of differential exosomal lncRNAs in LAA stroke. The figure shows 30 exosomal lncRNAs with the most significant differential expression (|log_2_fold change| ≥2, and *P* value < 0.05).

Three upregulated (exo‐lnc_000048, exo‐lnc_001350, exo‐lnc_016442) and two downregulated genes (exo‐lnc_002015, exo‐lnc_013144) (based on the fold change, FPKMs, and the function of target genes) were detected in an independent validation set (LAA = 40, control = 40) by qRT‐PCR, at the same time, ROC analysis was performed to preliminarily evaluate the potential diagnostic value of exosomal lncRNAs in LAA stroke. The sequences of specific primer were shown in Supplementary Table S8. As shown in Figures S2 and Table S3, the results, consistent with discovery set, indicated exosomal lncRNAs exhibited diagnostic performance in LAA stroke. To further confirm the reliability of above results, qRT‐PCR were furtherly performed in replication set. As shown in Figure 2, above exosomal lncRNAs were significantly upregulated (exo‐lnc_000048, exo‐lnc_001350, and exo‐lnc_016442) or downregulated (exo‐lnc_002015) in LAA group (*P *< 0.0001, Figures 2A‐D) except for exo‐lnc_013144 (*P *> 0.05, Figure 2E). To further confirm whether exosomal lncRNAs could distinguish LAA stroke from SAO stroke or AS patients, we increased the subgroups. The results indicated that the expression of upreglated exosomal lncRNAs were higher in LAA group than SAO, AS, and control groups (*P *< 0.001; after Bonferroni correction; Figures 2F‐H). Of note, exo‐lnc_013144 was significantly downregulated in AS group but not changes in LAA group (AS *vs*. control, *P *< 0.001; Figure 2J). Moreover, the levels of exo‐lnc_002015 was no difference between LAA and SAO groups (*P *> 0.05; Figure 2I). Eventually, exo‐lnc_000048, exo‐lnc_001350 and exo‐lnc_016442 (specifically and stably expressed in LAA group) were enrolled in Logistic regression analysis. The levels of exo‐lnc_000048, exo‐lnc_001350, and exo‐lnc_016442 remained significantly associated with increased odds of LAA stroke (Figure 2K). ROC demonstrated exo‐lnc_000048, exo‐lnc_001350, and exo‐lnc_0016442 exhibited AUCs of 0.829, 0.920, and 0.858, respectively (Figure S3A, Table S4). Furtherly, integrated exosomal lncRNAs panel performs better diagnostic ability than individual factor at predicting LAA stroke: combination of three exosomal lncRNAs exhibited an AUC of 0.936. (Figure 2L). Of note, the traditional biomarkers of TG, TC and LDL showed poor AUCs of 0.598, 0.611, and 0.541, respectively (Figure S3B, Table S4). The addition of traditional factors could not increase the diagnostic efficacy of exosomal lncRNAs (AUC: 0.936, Figures 2L, 2M).

**FIGURE 2:**
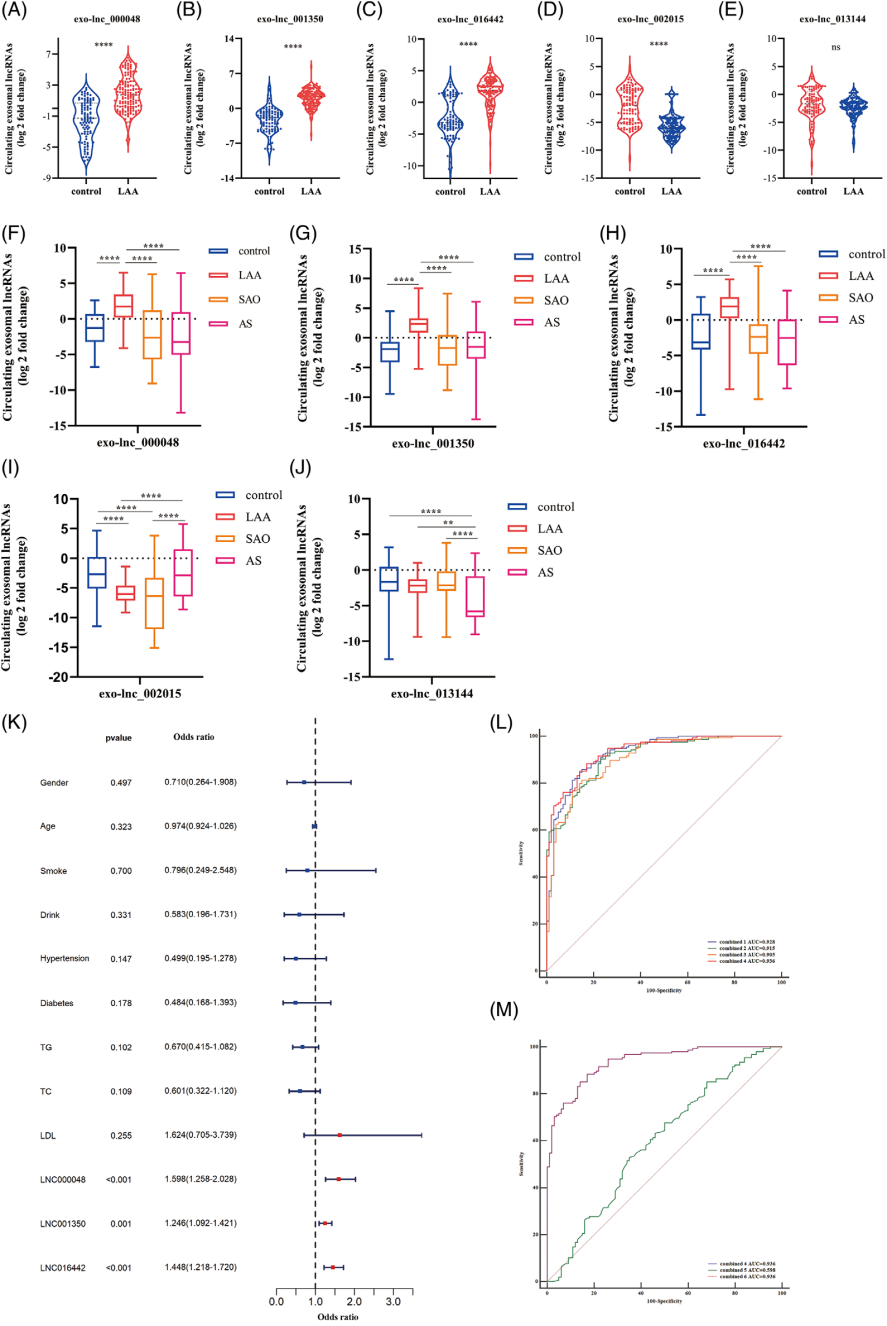
The analysis of selected exosomal lncRNAs in replication set. (A‐E) Expression of exo‐lnc_000048 (A), exo‐lnc_001350 (B), exo‐lnc_016442 (C), exo‐lnc_002015 (D) and exo‐lnc_013144 (E) in LAA stroke and control group by qRT‐PCR. ^ns^ *P *> 0.05, **** *P *< 0.0001. Mann‐Whitney U test. (F‐J) Expression of exo‐lnc_000048 (F), exo‐lnc_001350 (G), exo‐lnc_016442 (H), exo‐lnc_002015 (I), and exo‐lnc_013144 (J) in LAA stroke, SAO stroke, AS, and control by qRT‐PCR. * *P *< 0.05, ** *P *< 0. 001, **** *P *< 0.0001 by Kruskal‐Wallis test with Bonferroni corrected. (K) Odds ratio with 95% CI for the exosomal lncRNAs and risk factors in LAA stroke. (L) ROC analysis evaluates the diagnostic values of integrated exo‐lnc_000048, exo‐lnc_001350, and exo‐lnc_016442 for LAA stroke: combined 1: exo‐lnc_000048 plus exo‐lnc_001350; combined 2: exo‐lnc_000048 plus exo‐lnc_016442; combined 3: exo‐lnc_001350 plus exo‐lnc_016442; combined 4: exo‐lnc_000048 plus exo‐lnc_001350 plus exo‐lnc_016442. (M) ROC analysis evaluates the diagnostic values of integrated exo‐lnc_000048, exo‐lnc_001350, exo‐lnc_016442, TG, TC, and LDL for LAA stroke: combined 5: TG plus TC plus LDL; combined 6: combined 4 plus combined 5.

Traditional NIHSS and mRS scores are often used as severity and prognostic indicator for stroke. Here, we evaluated the relevance of exosomal lncRNAs, NIHSS and prognosis. Exo‐lnc_000048, exo‐lnc_001350 and exo‐lnc_016442 levels were elevated with the increase in the degree of severity of stroke (Figures 3A‐C). For prognosis, exo‐lnc_000048, exo‐lnc_001350, and exo‐lnc_016442 were elevated more in unfavorable outcome than favorable outcome (*P *< 0.0001; Figures 3D‐F, Table S5). When combining all upregulated exosomal lncRNAs and risk predictors (NIHSS, sex, age, TG, TC, and LDL) in a multivariate model, only NIHSS, exo‐lnc_001350, and exo‐lnc_016442 remained predictors of functional outcome (Table S6). In addition, exosomal lncRNAs had a significantly higher prognostic capacity than NIHSS. Interestingly, strongly improved discernment was obtained through integrated exosomal lncRNAs rather than only NIHSS in a discernment model for unfavorable outcome (NRI = 0.2222, *P *< 0.0001) (Figure 3G, Table S7).

**FIGURE 3:**
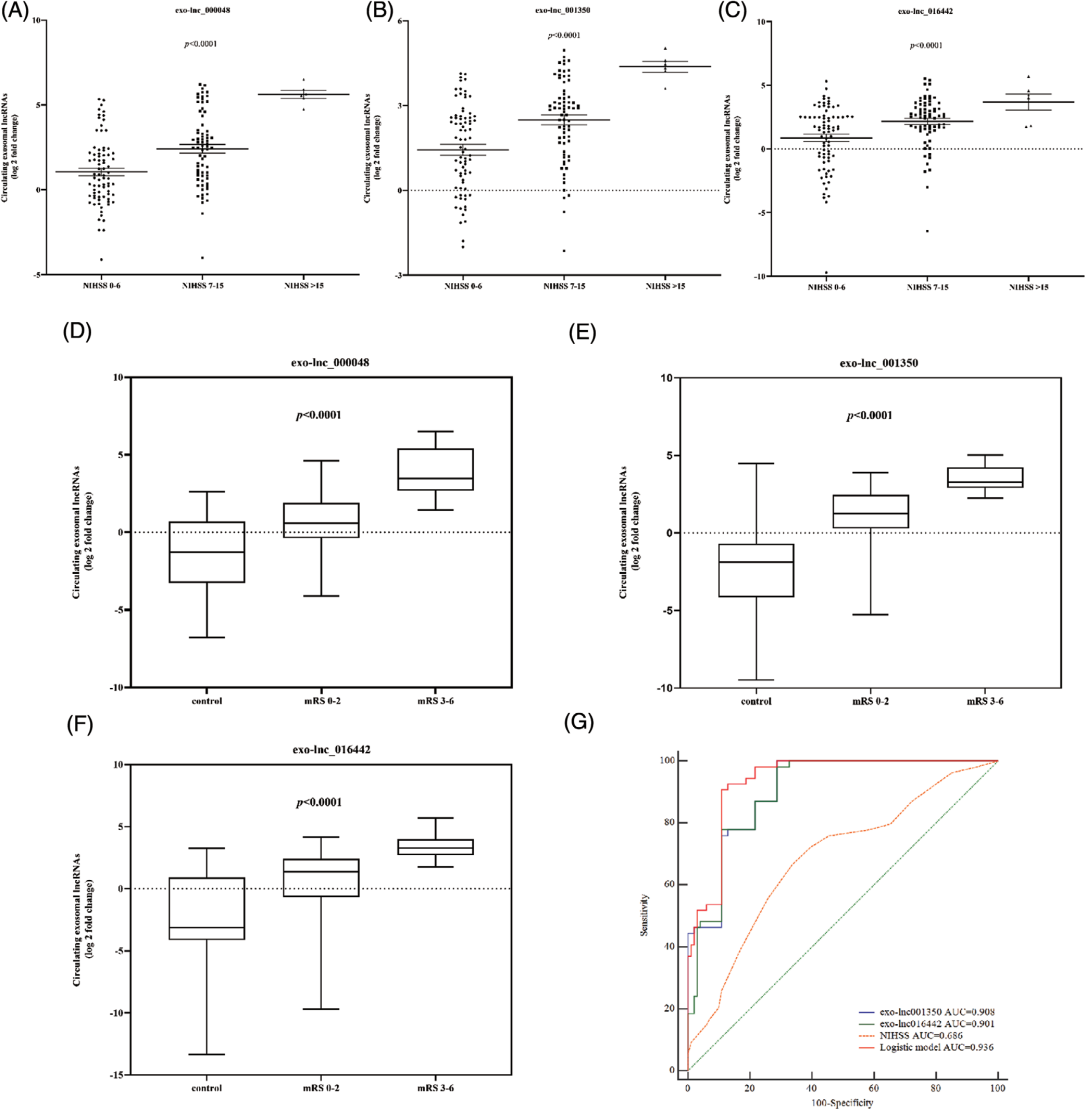
The ability of exosomal lncRNAs to predict the severity of stroke and functional outcomes. (A‐C) Exosomal lncRNAs levels and stroke severity. The relationship between exo‐lnc_000048 (A), exo‐lnc_001350 (B), exo‐lnc_016442 (C) levels and stroke severity. Kruskal‐Wallis test with Bonferroni corrected. (D‐F) The relative expression of exosomal lncRNAs in control and in different outcomes of LAA patients (according to 1‐month mRS: 0 to 2, > 2): exo‐lnc_000048 (D); exo‐lnc_001350 (B); exo‐lnc_016442 (F); Kruskal‐Wallis test with Bonferroni corrected. (G) ROC analysis evaluates the ability of exosomal lncRNAs and NIHSS for predicting functional outcome.

Although plasmatic lncRNAs are more readily available than exosomes, most of the lncRNAs in plasma exhibit poor stability due to nuclease degradation.^10^ To evaluate the expression of plasmatic lncRNAs, we further verified the expression of plasmatic lncRNAs in each group (Figures 4A‐E). The results revealed plasmatic lncRNAs exhibited unstable performance. Correlation analysis revealed that lncRNAs levels were poorly correlated between plasma and exosomes (Figure 4G), which indicted exosomal lncNRAs might be particular biomarkers for LAA stroke. Interestingly, we observed the AUCs of exosomal lncRNAs had an obvious advantage over plasmatic lncRNAs in identifying LAA stroke patients (lnc_000048: 0.825 *vs*. 0.591, lnc_001350: 0.920 *vs*. 0.584, lnc_016442: 0.858 *vs*. 0.706, Figure 4F)

**FIGURE 4:**
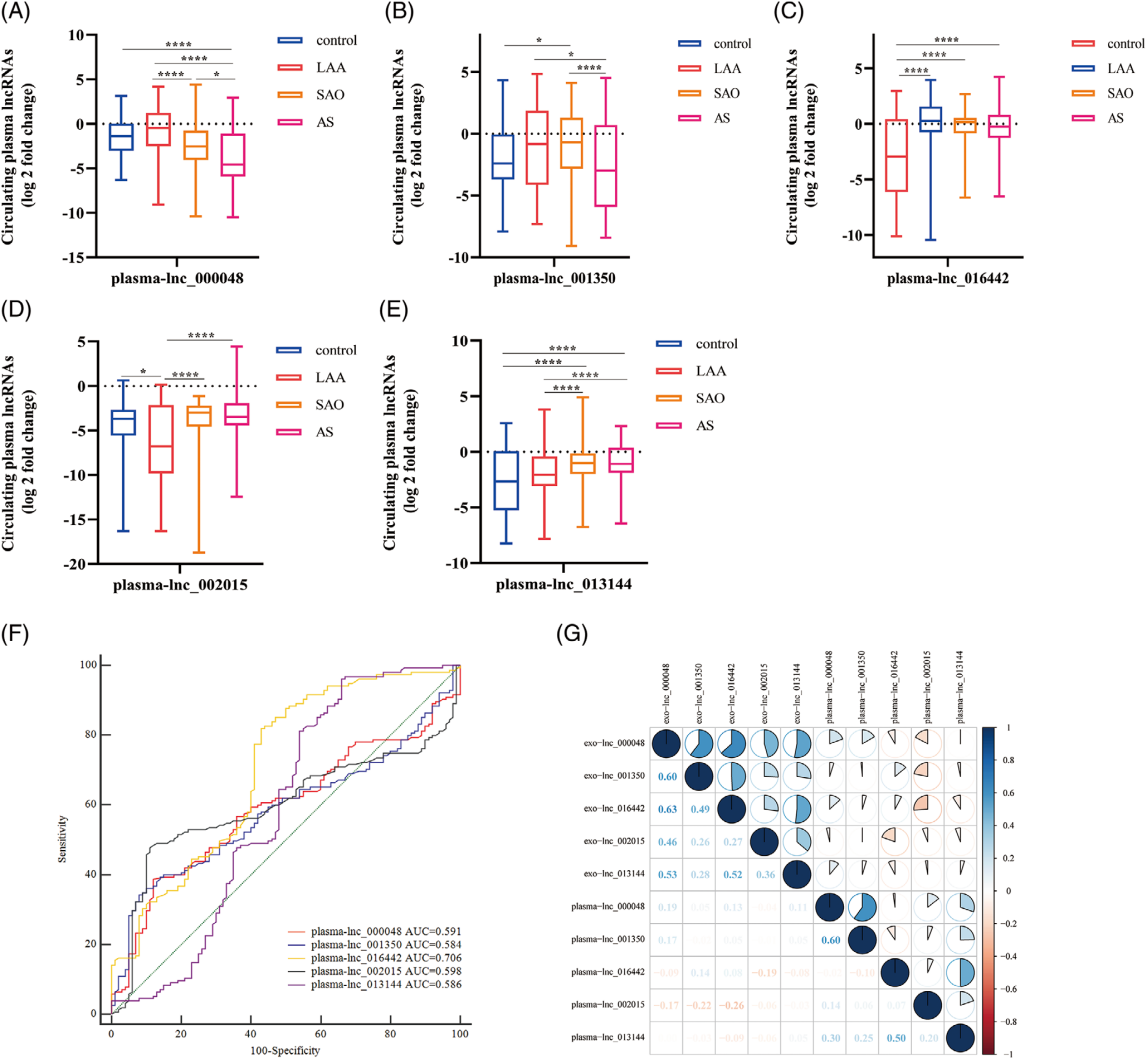
Analysis of the expression of lncRNAs in plasma. (A‐E) Expression of plasma‐ lnc_000048 (A), plasma‐lnc_001350 (B), plasma‐lnc_016442 (C), plasma‐lnc_002015 (D), and plasma‐lnc_013144 (E) in LAA stroke, SAO stroke, AS, and control groups by qRT‐PCR. *P < 0.05, ****P < 0.0001 by Kruskal‐Wallis test with Bonferroni corrected. (F) The ROC analysis to evaluate the diagnostic performance of plasmatic lncRNAs for LAA stroke. (G) Correlation analysis of lncRNAs levels between plasma and exosomes, blue represents a positive correlation, red represents a negative correlation, and the shade of the color and the proportion of the pie chart represent the correlation coefficient.

In conclusion, our study provided novel insights into the clinical value in exosomal ncRNAs for LAA stroke. We found that exosomal lncRNAs rather than plasmatic lncRNAs were significantly differential expressed in LAA strokes. Notably, combined exo‐lnc_000048, exo‐lnc_001350 and exo‐lnc_016442 exhibit better diagnostic performance. Additionally, exo‐lnc_001350 and exo‐lnc_016442 significantly elevated the prognostic capacity of NIHSS for unfavorable outcomes in LAA stroke, which indicted exosomal lncRNAs could be new and valuable biomarkers for the prognosis of LAA stroke. These findings suggested that exosomal lncRNAs might allow for better and earlier improved treatment strategies to effectively change the outcome of LAA stroke.

## CONFLICT OF INTEREST

The authors have no conflicts of interest.

## FUNDING INFORMATION

This work was supported by the National Natural Science Foundation of China (no. 81771259), and Natural Science Foundation of Shandong Province (ZR2020MH138).

## Supporting information

Supporting Information
