# Supplementary material for: Circulating exosomal lncRNAs as predictors of risk and unfavorable prognosis for large artery atherosclerotic stroke
Source: Clin Transl Med. 2021 Dec 19;11(12):e555. doi: 10.1002/ctm2.555 (PMC8684716; doi:10.1002/ctm2.555)
Supplement: Supplementary file 1 — Supporting Information [file CTM2-11-e555-s001.docx]

**SUPPLEMENTARY MATERIAL FOR LETTER**

**1. Supplementary Tables**

**TABLE S1** Baseline characteristics of the study population

| **Characteristics** | **Control** | **LAA** | **SAO** | **AS** | ***P*-value _all_** | ***P*-value_1_** | ***P*-value_2_** | ***P*-value_3_** |
| --- | --- | --- | --- | --- | --- | --- | --- | --- |
| **Age, years, mean (SD)** | 64.46 ±8.7 | 65.6 ±9.2 | 62.9 ±11.6 | 70.9 ±10.3 | <0.001 | 1.0 | 1.0 | <0.001 |
| **Male (%)** | 76(52.1) | 137(68.2) | 93(60) | 48(48) | 0.002 | 0.002 | 0.165 | 0.532 |
| **Smoker (%)** | 37(25.3) | 81(40.3) | 51(32.9) | 22(22) | 0.004 | 0.004 | 0.149 | 0.546 |
| **Alcohol consumers (%)** | 34(23.3) | 82(40.8) | 32(20.6) | 15(15) | <0.001 | 0.001 | 0.580 | 0.110 |
| **Hypertension (%)** | 83(56.8) | 132(65.7) | 99(63.9) | 65(65) | 0.361 | 0.095 | 0.213 | 0.20 |
| **Diabetes (%)** | 26(17.8) | 59(29.4) | 39(25.2) | 25(25) | 0.108 | 0.014 | 0.121 | 0.172 |
| **TG, median (IQI)** | 1.43(0.97) | 1.19 (0.62) | 1.32(0.91) | 1.24 (0.84) | 0.017 | 0.0122 | 1.0 | 0.459 |
| **TC, median (IQI)** | 4.56(1.29) | 4.23 (1.43) | 4.39(1.24) | 4.32(1.68) | 0.06 | 0.026 | 0.479 | 0.739 |
| **LDL, median (IQI)** | 2.56(0.98) | 2.49(1.38) | 2.65(0.89) | 2.78(1.21) | 0.099 | 0.887 | 0.099 | 0.054 |
| **Hypertension med use (%)** | 71(48.6) | 107(53.5) | 59(38.1) | 60(60) | 0.003 | 0.397 | 0.064 | 0.079 |
| **Diabetes med use (%)** | 27(18.5) | 44 (21.9) | 32(20.6) | 26(26) | 0.558 | 0.439 | 0.638 | 0.160 |
| **Anti-platelet drugs (%)** | 3(2.1) | 12(6) | 1(0.6) | 18(18) | <0.001 | 0.077 | 0.358 | <0.001 |
| **Lipid-lowing drug (%)** | 3(2.1) | 8(4) | 3(1.9) | 12(12) | <0.001 | 0.369 | 1.00 | 0.001 |

Continuous variables were expressed as mean ± standard deviation (SD) or median (interquartile range), Oneway ANOVA and Kruskal-Wallis were used for statistical analysis. Bonferroni correction was applied to multiple tests. Categorical values were presented as frequencies (percentages), Chi-square test was used for statistical analysis. _all_ Significance of the difference among groups; _1_Significance of the difference LAA cases and controls; _2_Significance of differences between SAO cases and controls; _3_Significance of the difference between AS cases and controls. Statistical significance was set at *P*<0.05. Abbreviations: LAA, large artery atherosclerotic; SAO, small artery occlusion; TG, triglycerides; TC, total cholesterol; LDL, low-density lipoprotein; AS, atherosclerosis.

**TABLE S2** Clinical scores in LAA group.

|  | **Favorable Outcome**  **(mRS 0–2)** | **Unfavorable Outcome**  **(mRS 3–6)** | ***P*-value** |
| --- | --- | --- | --- |
| **n** | 141 | 60 |  |
| **NIHSS** |  |  |  |
| **0-6** | 104 (88.1) | 14(11.9) | <0.001 |
| **7-15** | 36 (46.88) | 41(53.2) | <0.001 |
| **>15** | 1(16.7) | 5(83.3) | <0.001 |

Categorical values were presented as frequencies (percentages). The chi-square test was considered statistically significant at *P*< 0.05. **Abbreviations:** NIHSS, National Institutes of Health Stroke Scale; mRS, modified Rankin Scale

**TABLE S3** Results of relative expression of exo-lncRNAs in validation set in LAA stroke.

| **predictor** | **Relative expression (FC)** | **95%CI** | ***P*-value** |
| --- | --- | --- | --- |
| **exo-lnc_000048** | 3. 50 | 2.60-4.40 | <0.0001 |
| **exo-lnc_001350** | 3.35 | 2.14-4.56 | <0.0001 |
| **exo-lnc_016442** | 4.56 | 3.35-5.76 | <0.0001 |
| **exo-lnc_002015** | 0.35 | 0.19-0.52 | <0.0001 |
| **exo-lnc_013144** | 0.39 | 0.21-0.57 | 0.002 |

Statistical significance was set at *p* <0.05. Abbreviations: FC, fold change.

**TABLE S4** Receiver operator characteristic curve analysis of biomarker candidates in patients with LAA stroke.

| **predictor** | **AUC** | **95%CI** | ***P*-value** | **Sensitivity** | **Specificity** |
| --- | --- | --- | --- | --- | --- |
| **exo-lnc_000048** | 0.829 | 0.777-0.873 | <0.0001 | 93.55 | 55 |
| **exo-lnc_001350** | 0.920 | 0.879-0.950 | <0.0001 | 92.26 | 83 |
| **exo-lnc_016442** | 0.858 | 0.809-0.899 | <0.0001 | 93.55 | 64 |
| **TG** | 0.598 | 0.535-0.659 | 0.0083 | 52.9 | 65 |
| **TC** | 0.611 | 0.548-0.671 | 0.0021 | 46.45 | 74 |
| **LDL** | 0.541 | 0.477-0.603 | 0.2671 | 27.1 | 84 |
| **combined 1** | 0.928 | 0.889-0.956 | <0.0001 | 85.82 | 85 |
| **combined 2** | 0.915 | 0.874-0.946 | <0.0001 | 90.32 | 77 |
| **combined 3** | 0.905 | 0.862-0.938 | <0.0001 | 81.29 | 85 |
| **combined 4** | 0.936 | 0.898-0.962 | <0.0001 | 88.39 | 83 |
| **combined 5** | 0.598 | 0.535-0.659 | 0.0083 | 52.9 | 65 |
| **combined 6** | 0.936 | 0.898-0.962 | <0.0001 | 88.39 | 83 |

combined 1: exo-lnc_000048 plus exo-lnc_001350; combined 2: exo-lnc_000048 plus exo-lnc_016442; combined 3: exo-lnc_001350 plus exo-lnc_016442; combined 4: exo-lnc_000048 plus exo-lnc_001350 plus exo-lnc_016442; combined 5: TG plus TC plus LDL; combined 6: combined 4 plus combined 5. Statistical significance was set at *P* <0.05.

**TABLE S5** Baseline characteristics of exosomal lncRNAs and outcomes.

| **predictor** | **Favorable outcome** | | | **Unfavorable outcome** | | ***P*-value** |
| --- | --- | --- | --- | --- | --- | --- |
|  | Relative expression^*^ | | 95% CI | Relative expression | 95% CI |  |
| **exo-lnc_000048** | 3.61 | 2.52–4.70 | | 24.96 | 18.74–31.17 | p<0.0001 |
| **exo-lnc_001350** | 3.76 | 3.09–4.44 | | 13.13 | 11.10–15.16 | p<0.0001 |
| **exo-lnc_016442** | 3.40 | 2.64–4.15 | | 13.86 | 10.73–16.98 | p<0.0001 |

Th chi-square test was considered statistically significant at *P*< 0.05.

*Relative expression was exhibited by fold change.

**TABLE S6** Binary Logistic regression analysis for unfavorable outcome

| **predictor** | **Odd ratios** | **95%CI** | ***P*-value** |
| --- | --- | --- | --- |
| **exo-lnc_000048** | 0.917 | 0.793-1.061 | 0.245 |
| **exo-lnc_001350** | 1.607 | 1.088-2.373 | 0.017 |
| **exo-lnc_016442** | 1.153 | 1.037-1.281 | 0.008 |
| **NIHSS** | 1.153 | 1.004-1.324 | 0.043 |
| **TG** | 1.142 | 0.576-2.266 | 0.704 |
| **TC** | 1.340 | 0.692-2.596 | 0.385 |
| **LDL** | 0.891 | 0.373-2.129 | 0.891 |
| **Age** | 0.998 | 0.945-1.053 | 0.935 |
| **Gender** |  |  | 0.291 |

Statistical significance was set at *P*<0.05.

**TABLE S7** ROC analysis of unfavorable outcome

| **parameter** | **AUC** | **95% CI** | ***P*-value** |
| --- | --- | --- | --- |
| **NIHSS** | 0.686 | 0.597-0.775 |  |
| **exo-lnc_001350** | 0.908 | 0.864-0.951 | 0.0208 |
| **exo-lnc_016442** | 0.901 | 0.855-0.947 | 0.0120 |
| **Logistic model** | 0.936 | 0.900-0.972 | <0.0001 |

*p*-values indicate significance of AUC between parameters and NIHSS.

**Abbreviations:** ROC, receiver operating characteristic curve; AUC; Area Under Curve

**TABLE S8** Primer sequences in the study

| **Genes** | 5’-3’ | **Primer sequences** |
| --- | --- | --- |
| **lnc_000048** | Forward | TGGGCGGGATTCTGACTTAGAGG |
|  | Reverse | GGTGTATGTGCTTGGCTGAGGAG |
| **lnc_001350** | Forward | CCACCTCAACTGCCTGCCATG |
|  | Reverse | ACATCCGGCCTGCTCCTTCTC |
| **lnc_002015** | Forward | GCGTTCAGCACCATCACTTCTTTG |
|  | Reverse | CCTCCATCAACACCAAGCAGCAG |
| **lnc_013144** | Forward | TGAATACAGCCAGCTCTCCTCCTC |
|  | Reverse | AGGCGGACGAGTAGCGAAGAG |
| **lnc_016442** | Forward | CCTCTGTCTGTCTCTGTCCCTCTC |
|  | Reverse | CCCGACCAGGATGCCAGGAG |
| **ACTB** | Forward | GCGGACTATGACTTAGTTGCGTTACA |
|  | Reverse | TGCTGTCACCTTCACCGTTCCA |

**2. Supplementary Figures**

**
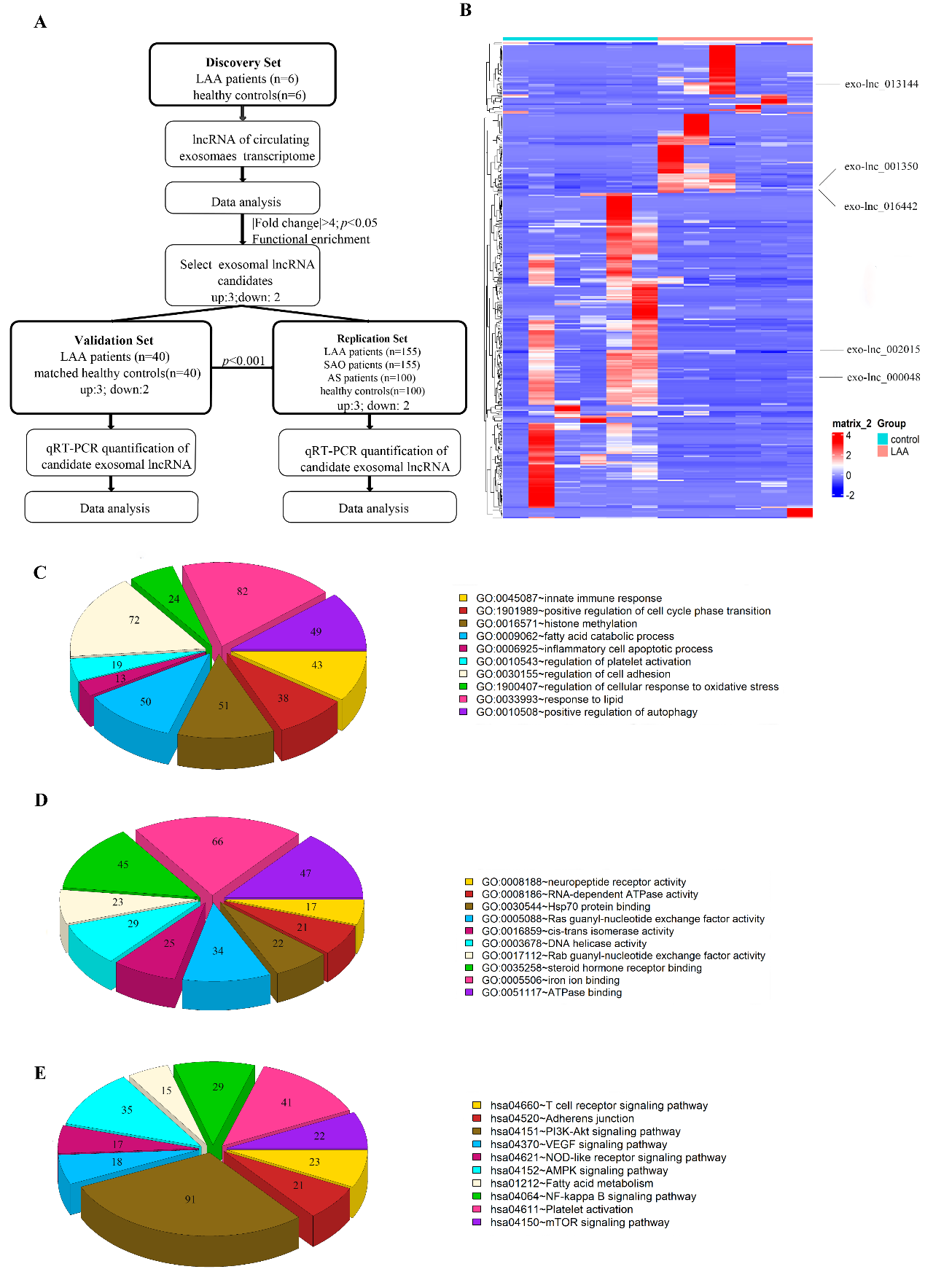
**

**FIGURE S1** The workflow of screening and functional analysis of differential expressed lncRNAs. (A)The flowchart illustrates 3 independent cohorts for discovery, validation, and replication sets. (B) A heatmap of the 319 DE exosomal lncRNAs across all 12 samples. Red represents upregulated genes, and blue represents downregulated genes. Five exosomal lncRNAs, which to be verified later, are displayed on the right of the heatmap. (C-E) Functional enrichment analysis of target genes of differential exosomal lncRNAs. The number of enriched genes in every item is shown on the pie chart: (C) Biological process analysis; (D) Molecular function analysis; (E) KEGG analysis. Abbreviations: DE gene, differentially expressed gene.


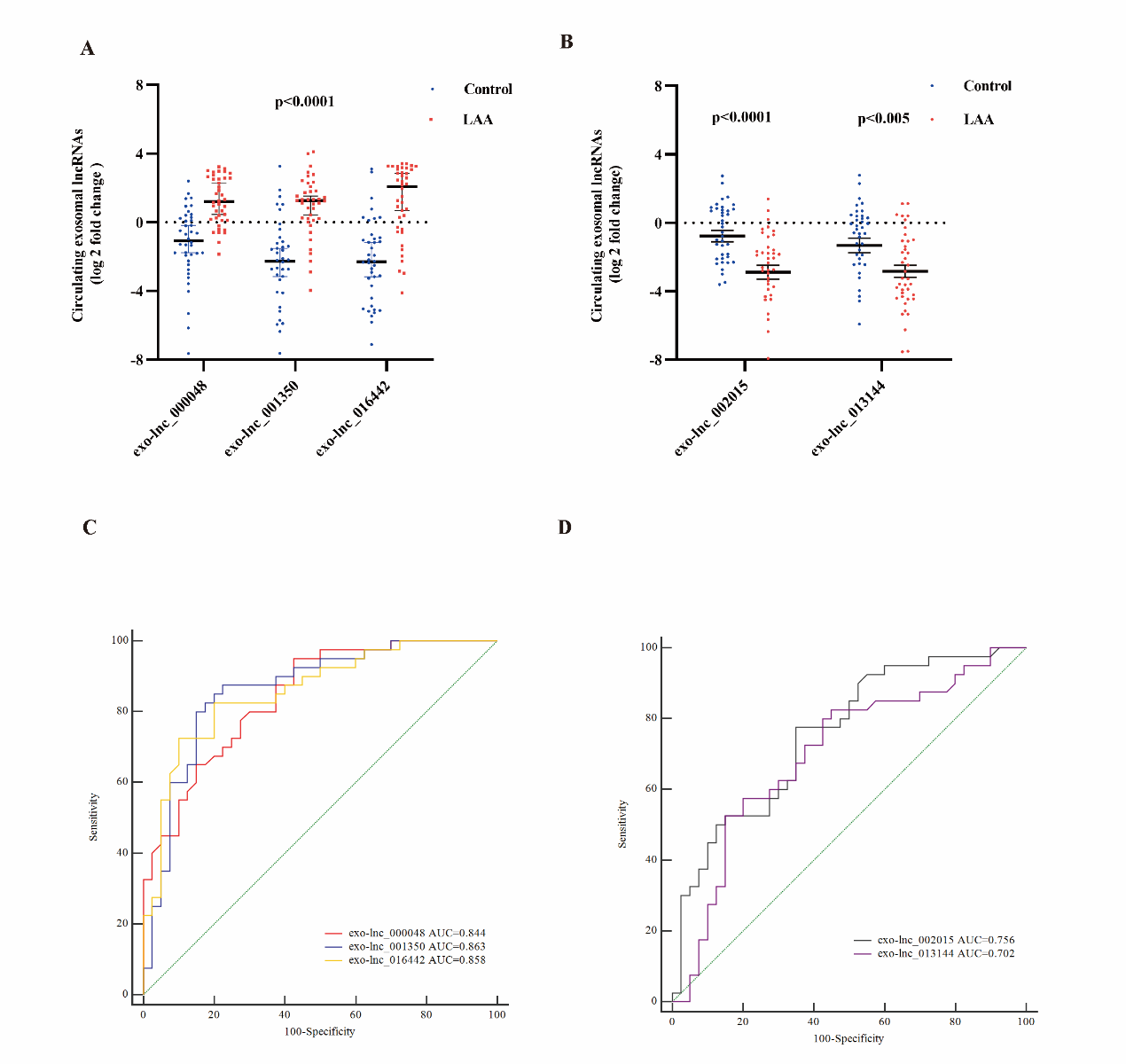


**FIGURE S2** The relative expression and AUCs of exosomal lncRNAs in validation set. (A-B) Validation of exo-lnc_000048, exo-lnc_001350, exo-lnc_016442, exo-lnc_002015 and exo-lnc_013144 by qRT-PCR (N=40/40, Control/LAA). (A) upregulated exosomal lncRNAs: exo-lnc_000048, exo-lnc_001350, and exo-lnc_016442; (B) downregulated exosomal lncRNAs: exo-lnc_002015 and exo-lnc_013144. Mann-Whitney test. (C-D) ROC were used to calculate AUCs of exosomal lncRNAs for differentiating LAA stroke patients and healthy control in validation set. (C) AUCs of upregulated exosomal lncRNAs; (D) AUCs of downregulated exosomal lncRNAs.


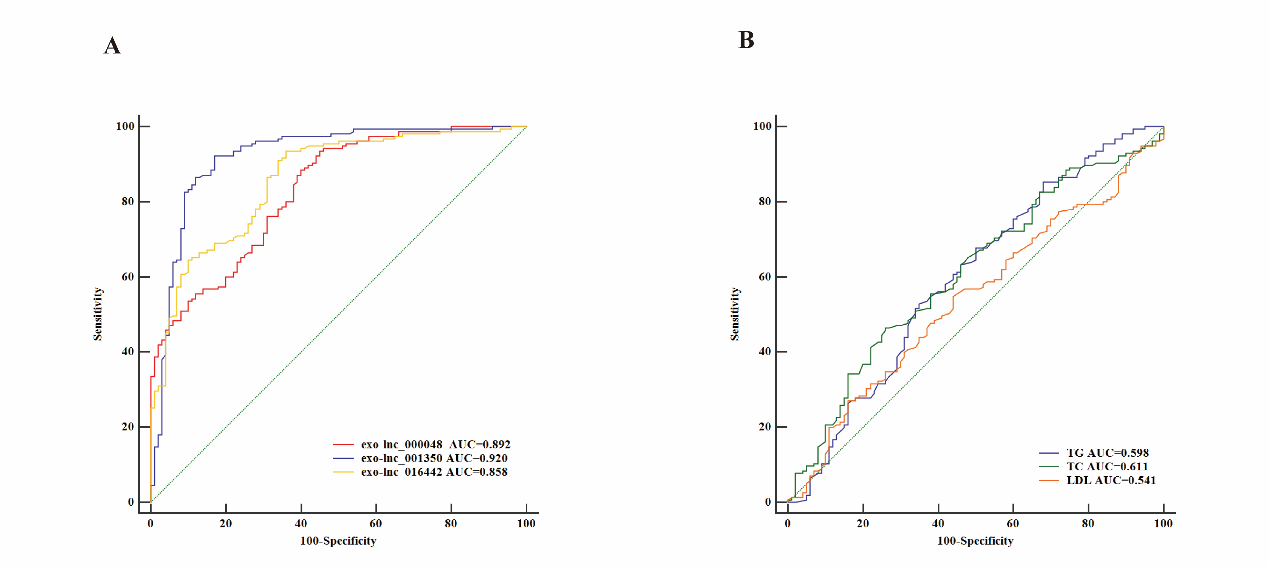


**FIGURE S3** ROC analysis of exosomal lncRNAs and clinical indicators.

(A) ROC analysis evaluates the diagnostic values of exo-lnc_000048, exo-lnc_001350, exo-lnc_016442 respectively for LAA stroke. (B) ROC analysis evaluates the diagnostic values of TG, TC, LDL for LAA stroke respectively.


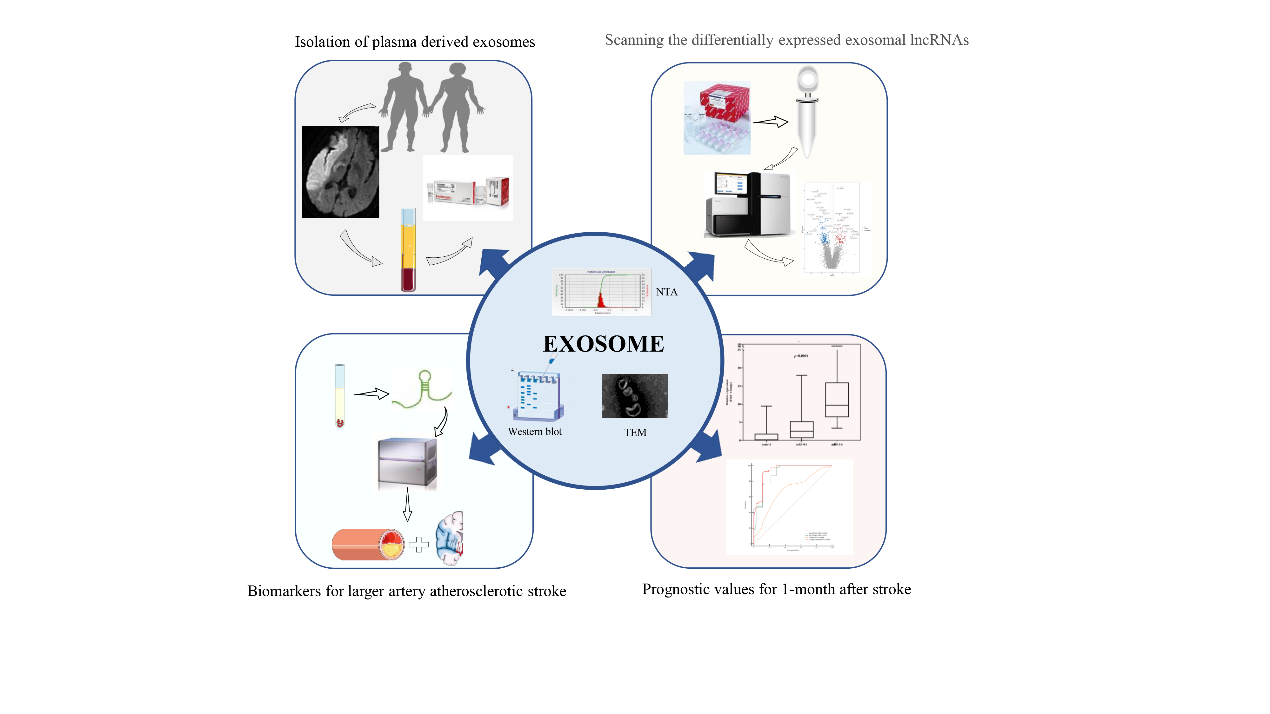


**FIGURE S4** All the workflow of the study.

**3. Supplementary Background**

Ischemic stroke has received increasing attention due to its high disability and mortality rates. Large artery atherosclerotic (LAA) stroke, the most common type of stroke according to the TOAST classification,^1^ has the worst prognosis and confers the heaviest family and social burden among all stroke subtypes.^2^ Accurate assessment of the etiology and disability risk of stroke can be used to inform clinical decisions and eventually improve the prognosis of stroke.^3, 4^ Although advances in neuroimaging have provided effective diagnostic strategies for stroke, the technique still has limitations, including lack abnormalities on imaging scan.^5, 6^ Similarly, clinical risk scores have rarely used to predict patient prognosis due to their complexity, lack of accuracy and effectiveness.^3, 7^ Therefore, identifying biomarkers that can quickly distinguish LAA stroke and predict outcomes is of great significance for exploring the potential values of clinical decisions. As protectors and carriers of RNA in vivo, exosomes have become stable and effective diagnostic targets in the study of diseases.

Exosomes, which are 30-150nm in diameter, are extensively enrichment in the blood and tissues.^8-10^ As a communicator among cells, exosomes assist multiple cellular content such as miRNAs, mRNAs, lncRNAs, proteins, and lipids to complete cell-to-cell transport and participate in various biological processes.^11-13^ Recent studies have found that the double membrane structure of exosomes can effectively protect the enclosed RNA from RNase in biological fluid; thus, exosomal RNAs are more reliable than those free floating in plasma.^8, 14-16^ In addition, exosomal RNAs can be used as a stable biomarker for disease progression assessment because of alterations in the process of cell differentiation and pathological changes.^17-19^ Currently, exosomal biomarkers related to stroke mostly focus on miRNAs and mRNAs. Our previous study found that exo-miR-145 is differentially expressed in ischemic stroke.^20^ LncRNAs, which regulate and affect the function and expression of miRNAs, mRNAs and proteins in tissue- and cell-specific manners, have attracted increasing attention as peripheral biomarkers for a variety of diseases due to their stable secondary structures and important biological functions. ^21, 22^

LncRNAs are classes of transcripts more than 200 nucleotides long with limited protein-coding potential that play a crucial part in the multilevel regulation of gene expression as transcriptional or post-transcriptional regulation.^23, 24^ LncRNAs can affect the occurrence and development of atherosclerosis by regulating endothelial cell migration, macrophage polarization, foam cell formation, and other biological processes, eventually leading to the occurrence of cardiovascular and cerebrovascular diseases.^25, 26^ Leisegang M et al. found that lncRNA MANTIS limits ICAM-1 mediated monocyte adhesion to endothelial cells, which potentially accelerate atherosclerosis development in humans.^27^ Cheryl Dykstra-Aiello et al. provided evidence of altered and sexually dimorphic lncRNA expression in peripheral blood and recommended that lncRNAs could be potential biomarker for stroke development.^28^ LncRNAs, as the regulator of genes, are involved in the occurrence and development of many diseases. Owing to the protection and intercellular transmission of exosomes, research on the mechanism of exosomal lncRNAs in atherosclerosis and stroke has become a promising topic.^29^ However, few studies have focused on exosomal lncRNAs as diagnostic and prognostic biomarkers in patients with LAA stroke.

In this study, we aimed to explore the differential expression profiles of exosomal lncRNAs and identify novel exosomal lncRNAs as diagnostic and prognostic biomarkers in LAA stroke. Further, we compared the diagnostic performance of exosomal lncRNAs and plasma lncRNAs.

**4. Supplementary Methods**

**4.1 Study population enrollment**

A total of 602 participants were recruited from May 1, 2019, to January 31, 2021, at the Affiliated Hospital of Qingdao University. Subtypes of ischemic stroke (IS) were determined according to the Trial of Org 10172 in Acute Stroke Treatment (TOAST).^1^ Ischemic stroke patients were classified into two groups: large-artery atherosclerosis (LAA, 137 males and 64 females) and small artery occlusion (SAO, 93 males and 62 females). We also recruited 100 patients with atherosclerosis (AS, 52 males and 48 females) and 146 health controls (control, 76 males and 70 females). The discovery samples included 6 LAA patients and 6 healthy controls. Validations were performed in 40 patients with LAA and 40 controls. The replication samples consisted of 155 LAA patients, 155 SAO patients, 100 AS patients and 100 healthy controls. The inclusion and exclusion criterion are shown as follows:^8, 30^

Inclusion criteria of stroke patients were applied: age ≥ 18 years; ischemic stroke within 72 hours after the event; LAA and SAO stroke patients were included according to TOAST classification criteria. AS patients were recruited as angiographically or carotid ultrasound verified 50 to 99 percent stenosis or occlusion of intracranial or extracranial artery without clinical symptoms. Control population were recruited from the Physical Examination Center. Exclusion criteria included nonatherosclerotic intracranial stenosis (e.g., moyamoya disease, vasculitis, or anatomy); potential sources of cardiac embolism (e.g., atrial fibrillation); incomplete cerebrovascular condition examination; and tumor or severe liver and kidney dysfunction. Systematic cerebrovascular examinations were performed on all participants. The correlation between the degree of atherosclerosis and stroke was determined by experienced neuroscientists. The atherosclerosis group was defined as the presence of atherosclerosis without the infarction and without the clinical manifestations of infarction.

This study was approved by the Ethics Committee of the Affiliated Hospital of Qingdao University.

**4.2 Clinical protocol**

A flow chart of the study is shown in **Figure S1A and Figure S4**.

National Institutes of Health Stroke Scale (NIHSS) was used to assess the neurological deficits on admission in all patients. Stroke severity was divided into 3 groups by NIHSS scores: Mild stroke was defined as an NIHSS score ranged 0-6, moderate stroke was defined as an NIHSS score ranged 7-15, and severe stroke was defined as an NIHSS score >15, which is consistent with the existing literature.^31^

The modified Rankin Scale (mRS) score was recorded at the 30-day follow-up. An mRS score of 0 to 2 was defined as a favorable outcome, whereas an mRS score >2 was defined as a poor outcome at one month after stroke.^3, 32^

**4.3 Baseline clinical data collection**

Baseline data, including demographic data and clinical information such as age, sex, alcoholism, smoking status, health history, and medications were collected from all participants. Traditional detection information, such as total cholesterol (TC), triglyceride (TG), and low-density lipoprotein (LDL) were also collected.

**4.4 Plasma processing**

Peripheral whole blood (5 ml) was collected from each participant and centrifuged at 3,000 × g at 4°C for 15 min within 3 h of collection. The plasma samples were stored at -80°C until analysis.

**4.5 Isolation of Exosomes**

Total plasma-derived exosomes were extracted and identified according to the methods recommended by International Society for Extracellular Vesicles.^33^ Exosomes were extracted using Total Exosome Isolation reagent (Invitrogen, Carlsbad, 4484450, USA) according to the manufacturer’s protocol.^34, 35^ Briefly, protease K (0.05 volumes) was added to the plasma and incubated at 37°C for 10 min. Exosome extraction reagent (0.2 volumes) was added to the mixture and incubated at 4°C for 30 min. The mixture was centrifuged at 10,000 × g for 5 min at room temperature. The supernatant was removed, and exosomes were resuspended and stored at -80°C for further detection.

**4.6 Nanoparticle tracking analysis**

Resuspended exosomes were examined by Nanotrac wave II (Microtrac, USA) equipped. Through a 3mW 780nm semiconductor fixed-position laser, the particle movement of exosomes as well as the size and quantity of particles were detected.

**4.7 Transmission electron microscopy**

The exosome suspension (20 µL) was deposited on a copper grid for 10min at room temperature and then washed with sterile distilled water. The cells were negatively stained with 2% uranyl acetate solution for one minute and dried for a few minutes at room temperature. The samples were observed and photographed using a transmission electron microscope (Thermo Fisher, USA).

**4.8 Western blot**

Exosomes were lysed using a mixture of RIPA and PMSF (Solarbio, USA). The BCA assay detected the total protein concentration. The lysate was heated to 95°C for 5 min and stored at -20°C. Protein samples were added to SDS polyacrylamide gels (25µg/lane) and then transferred to polyvinylidene fluoride membranes (Millipore, USA). Exosomes were identified using positive markers CD9, CD63, and TSG101 (Abcam, USA) as well as a negative marker, GRP94 (Abcam, USA). Proteins were visualized using a chemiluminescence system (Vilber, France).

**4.9 RNA extraction and Quality analyses**

Exosomal RNA and plasma RNA were isolated using the miRNeasy Serum/Plasma Advanced Kit (Qiagen, cat. 217204, Germany)^36^. The protocol was performed according to the manufacturer’s instructions. RNA integrity was tested using a Bioanalyzer 2100 system (Agilent Technologies, USA). The concentration of exosomal RNA is 20±2.8ng/μl, there was no difference among different samples.

**4.10 Library construction and sequencing**

Ribosomal RNA was eliminated using the Epicenter Ribo-zero™ rRNA Removal Kit (Epicenter, USA). First strand cDNA was generated using random hexamer primers and MuLV Reverse Transcriptase (RNaseH). Second-strand cDNA synthesis was subsequently performed using DNA Polymerase I and RNase H. Then, PCR was performed with Phusion High-Fidelity DNA polymerase, universal PCR primers and Index (X) Primer. Finally, the products were purified (AMPure XP system). Sequencing libraries were constructed using rRNA-depleted RNA using the NEBNext® Ultra™ Directional RNA Library Prep Kit for Illumina® (NEB, USA) according to the manufacturer’s recommendations and sequenced on an Illumina HiSeq 2500 platform, and 125 bp paired-end reads were generated. The specific process of sequencing is completed with the assistance of Novogene (Novogene, China).

**4.11 Differential expression analysis of exosomal lncRNAs**

Raw data (raw reads) in FASTQ format were first processed through in-house Perl scripts to obtain clean data (clean reads). At the same time, the Q20, Q30 and GC contents of the clean data were calculated. All downstream analyses were based on clean data with high quality. Eligible transcripts were obtained by matching, screening, and evaluation. The expression of exosomal lncRNAs was transformed into FPKMs based on standardized of library size and gene length by StringTie.^37,38^ The number of measurements was assessed using the Ballgown suite, Cufflinks, and edgeR. Transcripts with |log2fold change| ≥2, and *P*<0.05 were assigned as differentially expressed. The results were visualized as volcano and heat maps using the R package (pheatmap and ggrepel, R i386 3.6.3).

**4.12 GO and KEGG enrichment analysis**

Functional enrichment of the target genes of differentially exosomal lncRNAs, was performed by combining the DAVID and KOBAS databases. A threshold of FDR (corrected *P* value) < 0.05, was used to identify the significantly altered pathways. R package (ggplot2) was used to visualize the results.

**4.13 Quantitative real-time polymerase chainreaction (qRT-PCR)**

qRT-PCR was used to identify the selected lncRNAs. Plasma exosomes and RNA were extracted as previously described. Simply, RNA samples (60ng) were added to synthesize cDNA by Goldenstar™ RT6 cDNA Synthesis Kit (TSK302, TSINGKE) and then 2μl cDNA was used for qRT-PCR by 2×T5 Fast qPCR Mix (TSE301, TSINGKE). The sequences of specific primer were shown in **Supplementary Table S8**. LightCycler 480 II system were used to detect lncRNAs expression. Total RNA input was normalized using ACTB as an endogenous control.^39^ The relative expression of lncRNAs was calculated using the 2- ∆∆Ct method,^40, 41^ where ∆∆Ct = (Ct target – Ct reference) treated- (Ct target – Ct reference) control. Both relative expression data were transformed into a log2 base before further analysis.^42^

**4.14 Statistical analysis**

All statistical analyses were performed using SPSS (version 13.0; SPSS, USA) and GraphPad Prism 6.0 (GraphPad Software, USA). Continuous variables with normally distributed data are presented as mean ± standard deviation (SD), and non-normally distributed data as medians and interquartile range (25th and 75th percentiles). Categorical data were presented as counts and percentages. The Shapiro Wilks test was used to assess the normality of the distribution. ANOVA or Kruskal-Wallis followed by Dunn’s multiple comparison test were used for group-wise comparisons (n >2 groups). Mann Whitney or Student’s t-test was used for the two groups. For categorical variables, we used the Chi-squared test or Fisher’s exact test. All tests were performed 2-sided, and statistical significance was set at *P*<0.05. If indicated, a Bonferroni correction was applied for multiple tests.

Logistic regression analysis was used to assess the association between exosomal lncRNA expression and stroke risk factors. Odds ratios (ORs) with 95% confidence intervals were calculated. The continuous Net Reclassification Index was calculated using nricens package to compare multiple discrimination models.^43^ A Net Reclassification Index >0 was considered a positive improvement in discriminative capacity. Spearman’s rank correlation test or Pearson’s correlation test was performed as appropriate. The diagnostic and prognostic performances of the same exosomal lncRNA in LAA stroke were evaluated by receiver operating characteristic (ROC) curves; the area under the curve (AUC), sensitivity and specificity were calculated to compare the diagnostic performance of different exosomal lncRNAs (DeLong’s test). ^44-46^ R packages (pheatmap, ggplot2, nricens, corrplot) and MedCalc software were used to visualize the results.

**5. Supplementary Discussion**

This study comprehensively analyzed exosomal differentially expressed lncRNAs in LAA stroke. In this study, we established a set of circulating exosomal lncRNAs (exo-lnc_000048, exo-lnc_001350, and exo-lnc_016442) to show discrimination for LAA patients and controls. In addition, we found that the elevated expression of exo-lnc_000048, exo-lnc_001350, and exo-lnc_016442 were consistent with the severity of LAA stroke. Furthermore, we identified a relationship among unfavorable outcomes, exo-lnc_001350 and exo-lnc_016442. Importantly, exo-lnc_001350 and exo-lnc_016442 improved the prognostic value of the NIHSS score. Thus, we inferred that exosomal lncRNAs have potential clinical utility as diagnostic and prognostic biomarkers for LAA stroke.

In the present study, differentially expressed exosomal lncRNAs were identified and verified in LAA stroke patients and controls. Here we identified 3 novel exosomal lncRNAs (exo-lnc_000048, exo-lnc_001350, and exo-lnc_016442) as biomarker candidates for LAA stroke. Functional enrichment analysis of the target genes of these exosomal lncRNAs was carried out, the results showed differential exosomal lncRNAs were associated with atherosclerosis. For instance, KEGG: hsa04611, which represents platelet activation, has been proven to be related to atherosclerosis.^47-49^ Carresi et al. reported that platelet activation contributes to remodel of the vascular wall, plaque formation, vascular stenosis and obstruction.^50^ Therefore, we speculated that exosomal lncRNAs may influence the progression of LAA stroke. These exosomal lncRNAs were validated in the validation and replication sets. Interestingly, exo-lnc_000048, exo-lnc_001350, and exo-lnc_016442 were differentially expressed in LAA compared to control and in LAA compared to SAO, but not in SAO compared to control. These findings raised the possibility that the differences in exosomal lncRNAs reflected the underlying pathobiology of atherosclerotic cerebrovascular rather than acute ischemic stroke (AIS).

Notably, exo-lnc_000048 was differentially expressed in both LAA and AS compared to the control, but the difference was more pronounced in the LAA group. Considering the presence of atherosclerosis in both groups, the difference was in the occurrence or absence of stroke. Therefore, we speculate that exo-lnc_000048 may be related to plaque rupture in atherosclerosis. However, more imaging features are needed to support our inferences in the future.

Although there are many studies about the RNAs in exosomes, few studies have compared the RNA in plasmatic exosomes and in plasma. According to the study of Ling-Yun Lin on exosome lncRNA in early gastric cancer, the relative plasma levels of total circulating lncUEGC2 were down-regulated by approximately two-fold in plasma under RNase treatment (P = 0.0305), which proves that free lncRNA in plasma is easily degraded by nuclease.^16^ In our study, we found that the R^2^ (correlation coefficient) was varied dramatically from -0.02 to 0.63 in different lncRNAs. There was no significant correlation between exosomal lncRNAs and plasmatic lncRNAs, which may be related to the different origin of lncRNAs. Our results were consistent with those of Li Min in colon cancer.^51^ However, the mechanism underlying this phenomenon requires further research.

We also compared the diagnostic ability of LAA stroke between exosomal lncRNAs and plasmatic lncRNAs. Our results showed that the AUCs of exosomal lncRNAs were higher than those of plasma for lnc_000048, lnc_001350, and lnc_016442. To obtain higher diagnostic performance, we performed a strategy of combining metrics just like Takehito Shukuya did in the study of non-small-cell lung cancer.^52^ Eventually, we found that combining exosomal lncRNAs and traditional clinical indicators (TG, TC and LDL) together resulted in significant increase of AUCs in identifying LAA stroke than traditional clinical indicators alone. This phenomenon may provide new insights for the development of detection tools for LAA stroke patient.

NIHSS scores, a scale used to assess the severity of stroke, cannot be applied objectively in clinical practice due to the influence of the professional level of the operators and the different degrees of cooperation of the subjects.^3^ Katan discovered that MR-proANP concentrations were elevated with the increased severity of stroke, as defined by the NIHSS.^31^ To illustrate whether lncRNA expression levels correlated with stroke severity, we performed a subgroup analysis of exosomal lncRNAs and NIHSS scores in replicated set. We found that exo-lnc_000048, exo-lnc_001350 and exo-lnc_016442 significantly correlated with NIHSS, independent of other factors (TG, TC, LDL, age, and sex). Future validation of individual lncRNAs in a larger cohort is required.

In addition, almost one-third of patients with stroke die or experience disability within the first month.^3, 53^ Therefore, it is important to identify patients with poor prognosis to facilitate clinical decision-making and evaluation. Although there are some clinical scores that can predict the prognosis of stroke, they are difficult to fully use in clinical practice because of the complexity of the scale as well as the lack of precision and validation^3^. Identification of biomarkers that can quickly distinguish subtype of stroke and predict prognosis could improve patient management.^54^ In our study, unadjusted logistic regression analysis revealed that the levels of exosomal lncRNAs (exo-lnc_001350 and exo-lnc_016442) were predictors of one-month functional outcomes. In addition, NIHSS also reflected the functional prognosis, and the result was consistent with the study of the prognostic value of MR-pro-ANP in ischemic stroke in Switzerland.^31^ Adjusted logistic regression analysis revealed that the levels of exo-lnc_001350, exo-lnc_016442, and NIHSS were predictors of one-month functional outcomes independent of the presence of age, sex, TG, TC, and LDL. Our results showed that both exo-lnc_001350, exo-lnc_016442, and NIHSSS could independently predict functional prognosis. The combination of exo-lnc_001350, exo-lnc_016442, and NIHSS can achieve higher prognostic efficacy. The use of combined biomarkers for the prognosis of patients with stroke is well established. Our results indicate that combination of biomarkers and clinical parameters may be a reasonable and promising method for predicting the prognosis of stroke patients.

Our study had some limitations. The subjects were recruited from a single center and still need larger samples to verify the diagnostic and prognostic values of exosmal lncRNAs in LAA stroke. However, this study shows promising data for further multicenter trials including exosomal lncRNAs in a prognostic biomarker panel. Further, we will investigate and clarify the origin and target of exosomes as well as the potential functions of exosomal lncRNAs in the subsequent mechanism research to explore exosomal lncRNAs deeply in the future.

In conclusion, our study provides insights into the expression levels of exosomal lncRNAs in patients with LAA stroke. We found that the levels of exosomal lncRNAs were significantly differentially expressed in LAA strokes, and the combination of exosomal lncRNAs had a significant diagnostic value. Additionally, the results also showed that exosomal lncRNAs are new and valuable biomarkers for the prognosis of LAA stroke. These findings suggest that exosomal lncRNAs may allow for better and earlier improved treatment strategies to effectively change the outcome of stroke for the first time.

**6. Supplementary Reference**

1. Adams H, Bendixen B, Kappelle L*, et al.* Classification of subtype of acute ischemic stroke. Definitions for use in a multicenter clinical trial. TOAST. Trial of Org 10172 in Acute Stroke Treatment. *Stroke* 1993; **24**: 35-41.

2. Meschia J. Alpha-1 antitrypsin dysfunction and large artery stroke. *Proc Natl Acad Sci U S A* 2017; **114**: 3555-3557.

3. Raman K, O'Donnell M, Czlonkowska A*, et al.* Peripheral Blood MCEMP1 Gene Expression as a Biomarker for Stroke Prognosis. *Stroke* 2016; **47**: 652-658.

4. Zhong C, Zhu Z, Wang A*, et al.* Multiple biomarkers covering distinct pathways for predicting outcomes after ischemic stroke. *Neurology* 2019; **92**: e295-e304.

5. Tiedt S, Prestel M, Malik R*, et al.* RNA-Seq Identifies Circulating miR-125a-5p, miR-125b-5p, and miR-143-3p as Potential Biomarkers for Acute Ischemic Stroke. *Circ Res* 2017; **121**: 970-980.

6. Whiteley W, Tseng M, Sandercock P. Blood biomarkers in the diagnosis of ischemic stroke: a systematic review. *Stroke* 2008; **39**: 2902-2909.

7. Wang Z, Lin Y, Liu Y*, et al.* Serum Uric Acid Levels and Outcomes After Acute Ischemic Stroke. *Mol Neurobiol* 2016; **53**: 1753-1759.

8. Pan S, Pei L, Zhang A*, et al.* Passion fruit-like exosome-PMA/Au-BSA@Ce6 nanovehicles for real-time fluorescence imaging and enhanced targeted photodynamic therapy with deep penetration and superior retention behavior in tumor. *Biomaterials* 2020; **230**: 119606.

9. Wang L, Bruce T, Huang S, Marcus R. Isolation and quantitation of exosomes isolated from human plasma via hydrophobic interaction chromatography using a polyester, capillary-channeled polymer fiber phase. *Anal Chim Acta* 2019; **1082**: 186-193.

10. Zheng X, Chen F, Zhang Q*, et al.* Salivary exosomal PSMA7: a promising biomarker of inflammatory bowel disease. *Protein Cell* 2017; **8**: 686-695.

11. Hosseini R, Asef-Kabiri L, Yousefi H*, et al.* The roles of tumor-derived exosomes in altered differentiation, maturation and function of dendritic cells. *Mol Cancer* 2021; **20**: 83.

12. Xiong H, Huang Z, Yang Z*, et al.* Recent Progress in Detection and Profiling of Cancer Cell-Derived Exosomes. *Small (Weinheim an der Bergstrasse, Germany)* 2021: e2007971.

13. You D, Lim G, Kwon S*, et al.* Metabolically engineered stem cell-derived exosomes to regulate macrophage heterogeneity in rheumatoid arthritis. *Science advances* 2021; **7**.

14. Beckham C, Olsen J, Yin P*, et al.* Bladder cancer exosomes contain EDIL-3/Del1 and facilitate cancer progression. *The Journal of urology* 2014; **192**: 583-592.

15. Théry C. Cancer: Diagnosis by extracellular vesicles. *Nature* 2015; **523**: 161-162.

16. Lin L, Yang L, Zeng Q*, et al.* Tumor-originated exosomal lncUEGC1 as a circulating biomarker for early-stage gastric cancer. *Mol Cancer* 2018; **17**: 84.

17. Guo X, Lv X, Ru Y*, et al.* Circulating Exosomal Gastric Cancer-Associated Long Noncoding RNA1 as a Biomarker for Early Detection and Monitoring Progression of Gastric Cancer: A Multiphase Study. *JAMA surgery* 2020; **155**: 572-579.

18. Xie M, Yu T, Jing X*, et al.* Exosomal circSHKBP1 promotes gastric cancer progression via regulating the miR-582-3p/HUR/VEGF axis and suppressing HSP90 degradation. *Mol Cancer* 2020; **19**: 112.

19. Hu W, Liu C, Bi Z*, et al.* Comprehensive landscape of extracellular vesicle-derived RNAs in cancer initiation, progression, metastasis and cancer immunology. *Mol Cancer* 2020; **19**: 102.

20. Yang W, Yin R, Zhu X*, et al.* Mesenchymal stem-cell-derived exosomal miR-145 inhibits atherosclerosis by targeting JAM-A. *Molecular therapy Nucleic acids* 2021; **23**: 119-131.

21. Qu L, Ding J, Chen C*, et al.* Exosome-Transmitted lncARSR Promotes Sunitinib Resistance in Renal Cancer by Acting as a Competing Endogenous RNA. *Cancer Cell* 2016; **29**: 653-668.

22. Tan S, Pastori C, Penas C*, et al.* Serum long noncoding RNA HOTAIR as a novel diagnostic and prognostic biomarker in glioblastoma multiforme. *Mol Cancer* 2018; **17**: 74.

23. Zheng R, Du M, Wang X*, et al.* Exosome-transmitted long non-coding RNA PTENP1 suppresses bladder cancer progression. *Mol Cancer* 2018; **17**: 143.

24. Kim S, Lim K, Yang S, Joo J. Long non-coding RNAs in brain tumors: roles and potential as therapeutic targets. *J Hematol Oncol* 2021; **14**: 77.

25. Simion V, Zhou H, Haemmig S*, et al.* A macrophage-specific lncRNA regulates apoptosis and atherosclerosis by tethering HuR in the nucleus. *Nat Commun* 2020; **11**: 6135.

26. Sallam T, Sandhu J, Tontonoz P. Long Noncoding RNA Discovery in Cardiovascular Disease: Decoding Form to Function. *Circ Res* 2018; **122**: 155-166.

27. Leisegang M, Bibli S, Günther S*, et al.* Pleiotropic effects of laminar flow and statins depend on the Krüppel-like factor-induced lncRNA MANTIS. *Eur Heart J* 2019; **40**: 2523-2533.

28. Dykstra-Aiello C, Jickling G, Ander B*, et al.* Altered Expression of Long Noncoding RNAs in Blood After Ischemic Stroke and Proximity to Putative Stroke Risk Loci. *Stroke* 2016; **47**: 2896-2903.

29. Cao Q, Guo Z, Yan Y, Wu J, Song C. Exosomal long noncoding RNAs in aging and age-related diseases. *IUBMB Life* 2019; **71**: 1846-1856.

30. Gottesman R, Mosley T, Knopman D*, et al.* Association of Intracranial Atherosclerotic Disease With Brain β-Amyloid Deposition: Secondary Analysis of the ARIC Study. *JAMA neurology* 2020; **77**: 350-357.

31. Katan M, Fluri F, Schuetz P*, et al.* Midregional pro-atrial natriuretic peptide and outcome in patients with acute ischemic stroke. *J Am Coll Cardiol* 2010; **56**: 1045-1053.

32. Chamorro A, Amaro S, Castellanos M*, et al.* Safety and efficacy of uric acid in patients with acute stroke (URICO-ICTUS): a randomised, double-blind phase 2b/3 trial. *The Lancet Neurology* 2014; **13**: 453-460.

33. Théry C, Witwer K, Aikawa E*, et al.* Minimal information for studies of extracellular vesicles 2018 (MISEV2018): a position statement of the International Society for Extracellular Vesicles and update of the MISEV2014 guidelines. *Journal of extracellular vesicles* 2018; **7**: 1535750.

34. Royo F, Zuñiga-Garcia P, Sanchez-Mosquera P*, et al.* Different EV enrichment methods suitable for clinical settings yield different subpopulations of urinary extracellular vesicles from human samples. *Journal of extracellular vesicles* 2016; **5**: 29497.

35. Morris D, Jaehne A, Chopp M*, et al.* Proteomic Profiles of Exosomes of Septic Patients Presenting to the Emergency Department Compared to Healthy Controls. *Journal of clinical medicine* 2020; **9**.

36. Androvic, P., Romanyuk, N., Urdzikova-Machova, L., *et al*. Two-tailed RT-qPCR panel for quality control of circulating microRNA studies. *Scientific Reports* 2019; **9**, 4255.

37. Liu, X., Wu, Z., Li, J., *et al*. Genome-Wide Association Study and Transcriptome Differential Expression Analysis of the Feather Rate in Shouguang Chickens. *Frontiers in Genetics* 2020; **11**, 613078.

38. Yan XM, Zhang Z, Liu JB, *et al*. Genome-wide identification and analysis of long noncoding RNAs in longissimus muscle tissue from Kazakh cattle and Xinjiang brown cattle. *ASIAN-AUSTRALASIAN JOURNAL OF ANIMAL SCIENCES* 2020.

39. Sasaki T, Lian S, Khan A, *et al*. Autolysosome biogenesis and developmental senescence are regulated by both Spns1 and v-ATPase. *Autophagy* 2017;**13**, 386-403.

40. Pfaffl M. A new mathematical model for relative quantification in real-time RT-PCR. *Nucleic Acids Res* 2001; **29**: e45.

41. Livak K, Schmittgen T. Analysis of relative gene expression data using real-time quantitative PCR and the 2(-Delta Delta C(T)) Method. *Methods (San Diego, Calif)* 2001; **25**: 402-408.

42. Shin MK, Kim J, Kim D, *et al*. Long non-coding RNAs are significantly associated with prognosis and response to therapies in gastric cancer. *Clinical and Translational Medicine* 2021; **11**, e421.

43. Pandey A, Mehta A, Paluch A*, et al.* Performance of the American Heart Association/American College of Cardiology Pooled Cohort Equations to Estimate Atherosclerotic Cardiovascular Disease Risk by Self-reported Physical Activity Levels. *JAMA cardiology* 2021.

44. DeLong, ER, DeLong, DM, *et al*. Comparing the areas under two or more correlated receiver operating characteristic curves: a nonparametric approach. *BIOMETRICS* 1988; **44**: 837-845.

45. Molodianovitch K, Faraggi D, Reiser B. Comparing the areas under two correlated ROC curves: parametric and non-parametric approaches. *Biomedical Journal* 2006;**48**: 745-757.

46. Yin J, Liu H, Liu Z, *et al*. Genetic variants in fanconi anemia pathway genes BRCA2 and FANCA predict melanoma survival. *JOURNAL OF INVESTIGATIVE DERMATOLOGY* 2015; **135**,:542-550.

47. Yeung J, Li W, Holinstat M. Platelet Signaling and Disease: Targeted Therapy for Thrombosis and Other Related Diseases. *Pharmacol Rev* 2018; **70**: 526-548.

48. Oggero S, de Gaetano M, Marcone S*, et al.* Extracellular vesicles from monocyte/platelet aggregates modulate human atherosclerotic plaque reactivity. *Journal of extracellular vesicles* 2021; **10**: 12084.

49. Becker R, Sexton T, Smyth S. Translational Implications of Platelets as Vascular First Responders. *Circ Res* 2018; **122**: 506-522.

50. Carresi C, Scicchitano M, Scarano F*, et al.* The Potential Properties of Natural Compounds in Cardiac Stem Cell Activation: Their Role in Myocardial Regeneration. *Nutrients* 2021; **13**.

51. Min L, Zhu S, Chen L*, et al.* Evaluation of circulating small extracellular vesicles derived miRNAs as biomarkers of early colon cancer: a comparison with plasma total miRNAs. *Journal of extracellular vesicles* 2019; **8**: 1643670.

52. Shukuya T, Ghai V, Amann J*, et al.* Circulating MicroRNAs and Extracellular Vesicle-Containing MicroRNAs as Response Biomarkers of Anti-programmed Cell Death Protein 1 or Programmed Death-Ligand 1 Therapy in NSCLC. *Journal of thoracic oncology : official publication of the International Association for the Study of Lung Cancer* 2020; **15**: 1773-1781.

53. Donnan G, Fisher M, Macleod M, Davis S. Stroke. *Lancet (London, England)* 2008; **371**: 1612-1623.

54. Harshfield E, Sims M, Traylor M, Ouwehand W, Markus H. The role of haematological traits in risk of ischaemic stroke and its subtypes. *Brain : a journal of neurology* 2020; **143**: 210-221.
